# Prevalence of Methicillin-Resistant *Staphylococcus aureus* and Associated Risk Factors among Patients with Wound Infection at Referral Hospital, Northeast Ethiopia

**DOI:** 10.1155/2020/3168325

**Published:** 2020-05-24

**Authors:** Yeterefwork Tsige, Senait Tadesse, Tsehaynesh G/Eyesus, Mulugeta Mihrete Tefera, Anteneh Amsalu, Marta Alemayhu Menberu, Baye Gelaw

**Affiliations:** ^1^Dessie Regional Health Research Laboratory, Dessie, Ethiopia; ^2^Medical Laboratory Department, Health Science College, Bahirdar University, Bahirdar, Ethiopia; ^3^Amhara Public Health Institute, Bahirdar, Ethiopia; ^4^Pharmacy Department, Bahirdar Health Science College, Bahirdar, Ethiopia; ^5^University of Gondar, Colleges of Medicine and Health Sciences, Gondar, Ethiopia

## Abstract

**Background:**

The spectrums of infections due to methicillin-resistant *Staphylococcus aureus* are manifold and are associated with worse outcomes. A study on the prevalence of these pathogens and their sensitivity patterns will give updated information which is very helpful for health personnel responsible in the management of patients and timely monitoring of the emergence of resistant bacteria. Hence, the study aimed at assessing the prevalence of methicillin-resistant *Staphylococcus aureus* and associated factors among patients with wound infection at Dessie Referral Hospital.

**Method:**

A cross-sectional study was conducted among 266 patients at Dessie Referral Hospital from February to May 2016. Wound swab samples were collected aseptically using Levine's technique and transported to Dessie Regional Laboratory by using brain-heart infusion transport media. Isolation of *Staphylococcus aureus* was done based on cultural and biochemical profiles. Drug susceptibility test was performed using the disc diffusion technique as per the standard and interpreted based on the Clinical and Laboratory Standards Institute guidelines. The data were entered and analyzed by using SPSS version 20.

**Result:**

*Staphylococcus* isolates from 266 processed wound swabs were 92 (34.58%). Of these, 26 (28.3%) were identified as methicillin-resistant *S. aureus* and 66 (71.7%) were methicillin-sensitive *S. aureus*. The overall prevalence of methicillin-resistant *S. aureus* among the study population was 9.8%. The isolated methicillin-resistant *S. aureus* showed full resistance to penicillin (100%) followed by erythromycin and ciprofloxacin (16, 61.5%) and cotrimoxazole and gentamicin (14, 53.8%). From the total *S. aureus* isolates, 20 (21.7%) of them showed multidrug resistance. Of these methicillin-resistant *S. aureus*, 18 (69.8%) showed high multidrug resistance. Patients who are farmers in occupation (AOR = 6.1, 95% CI (1.086–33.724)), admitted in the hospital (AOR = 3.56, 95% CI (1.429–8.857)), and have low BMI (<18.5) (AOR = 13.89, 95% CI (4.919–39.192)) were among the risk factors significantly associated with wound infection due to methicillin-resistant *S. aureus*.

**Conclusion:**

All methicillin-resistant *S. aureus* isolates were 100% resistant to penicillin and showed high multidrug resistance. Therefore, antibiotic susceptibility test should be performed prior to treatment.

## 1. Background

Wound is a break in the skin and exposes the underlying tissue to the outside environment. Loss of skin integrity by wounding provides a moist, warm, and nutritious environment for microbial colonization, proliferation, and infection [1, 2]. Common bacterial skin infections include *Staphylococcus aureus*, *Escherichia coli, Pseudomonas aeruginosa*, *Klebsiella pneumoniae*, *Streptococcus pyogenes*, *Proteus* species, *Streptococcus* species, and *Enterococcus* species [3, 4]. Among the most frequent members of wound infection, *Staphylococcus aureus* [5] is a leading cause of nosocomial infections (NI) and surgical wound infections [6]. It develops resistance to many antibiotics in recent years.

Methicillin-resistant *S. aureus* acquires its resistance via the methicillin resistance gene *mecA*, which encodes a low-affinity penicillin-binding protein (PBP2a) that is absent in susceptible *S. aureus* strains [7, 8]. This resistant penicillin-binding protein receptor does not bind well to most *β*-lactams and therefore allows MRSA to grow in their presence [8]. Methicillin-resistant *S. aureus* strains were recently classified as two groups by epidemiologic as well as molecular characteristics, namely, community-associated (CA) MRSA and healthcare-associated (HA) MRSA. Community-associated MRSA isolates are usually less resistant than HA-MRSA isolates [9].

Methicillin-resistant *S. aureus* is a major problem worldwide causing hospital-acquired infections [10]. It is estimated that MRSA infections within the healthcare setting alone affected more than 150,000 patients annually in the European Union, with an additional cost of 380 million euros [11]. The widespread and prolonged use of antibiotics leads to the emergence of resistant bacterial pathogens during wound infections contributing to high morbidity and mortality [12]. The spectrums of infections due to MRSA are manifold [13, 14] and are associated with worse outcome in addition to prolonged hospital stay, higher cost of treatment, and increased mortality [15, 16].

In some parts of Africa, 80% of *S. aureus* infection was resistant to methicillin, rendering treatment with standard antibiotics ineffective [17]. Even though different studies across the region in Ethiopia showed that the burden of MRSA constitutes a major public health problem [18–20], prevention and control strategies are not well established to minimize MRSA. In addition, antibiotics are widely and inappropriately used results in the increased prevalence of drug resistance strain bacteria such as MRSA, so that a study on the prevalence of these pathogens and their sensitivity patterns in healthcare facility will give updated information which is very helpful for health personnel responsible in the management of patients and timely monitoring of the emergence of resistant bacteria. In general, the current study results can also be used as input data to establish a guideline to minimize the burden of MRSA.

## 2. Methods

A cross-sectional study was conducted from February 2016 to April 2016 to assess the antibiotic resistance pattern of methicillin-resistant *Staphylococcus aureus* isolated from wound infection and associated risk factors in Dessie Referral Hospital (DRH). Dessie Referral Hospital is found in Dessie town with a distance of 400 km from the capital city of the county Addis Ababa and 471 km far from Bahir Dar which is the capital city of Amhara regional state. In Dessie town, there are one referral hospital, three private general hospitals, three health centers, five higher private clinics, and one regional health research laboratory where culture and susceptibility tests are performed. All patients suspected of wound infections and who have not taken antibiotics for the last two weeks prior to the study period were included in this study. The sample size was determined using a single population proportion formula at 19.6% [18] and 95% confidence interval (CI), and the total sample size was 266.

Sociodemographic related data and associated risk factors were collected by using a structured questionnaire.

### 2.1. Sample Collection and Processing

Wound samples were collected using Levine's technique [21]. The wound surface was cleaned with sterile gauze moistened with 70% alcohol. Dressed wounds were cleansed with sterile normal saline after removing the dressing. Aseptically, the end of a sterile cotton-tipped applicator was rotated over 1 cm^2^ area for 5 seconds with sufficient pressure to express fluid and bacteria to surface from within the wound tissue as technique stated by Levine and Gardner [22]. Samples from closed wound were collected after the skin was cleansed with 70% alcohol. Double wound swabs were taken from each wound at a point in time to increase the chance of recovering bacterial pathogens. All collected specimens were labeled and transported by using brain-heart infusion transport media to Dessie Regional Health Research Laboratory for culturing and antimicrobial susceptibility testing within 1 hour. Each wound specimen was inoculated on blood (Oxoid, Ltd., Basingstoke, Hampshire, England) and subcultured on mannitol salt agar. All plates were incubated in aerobic atmosphere at 35–37°C for 24 h.

*Staphylococcus aureus* was identified based on Gram-positive cocci in clusters, *β*-hemolytic colonies on blood agar, catalase and coagulase production, and yellow colony surrounded by yellow zone on mannitol salt agar [21].

### 2.2. Antimicrobial Susceptibility Test

Antimicrobial susceptibility test was carried out on each bacterial isolate using the disc diffusion method on Muller Hinton agar (MHA). Three to five pure colonies of each bacterium were picked and transferred to a tube containing 5 ml sterile nutrient broth. The preparation was mixed thoroughly to make the suspension homogeneous. The suspension was incubated at 37°C until the turbidity of the suspension adjusted to a 0.5 McFarland turbidity standard (bacterial concentration of 1.5 × 10^8^ colony-forming unit/ml) [23]. A sterile swab was dipped in the suspension, and the entire surface of the MHA plates was uniformly flooded with the suspensions and allowed to dry for about 15–30 minutes.

The antimicrobial impregnated disks were placed on the media using sterile forceps in such a way that each disk was placed at least 24 mm away from each other to avoid the overlapping zone of inhibition. After the disk was placed on the inoculated media, the plates were allowed to stand for 30 minutes so that the antibiotic will diffuse into the media. The plates were inverted and incubated at 35 ± 2°C for 24 h and observed for the zone of inhibition.

The selected antibiotic disks used were (Oxoid UK) penicillin (10IU), ciprofloxacillin (5 *μ*g), cotrimoxazole (1.25/23.75 *μ*g), doxycycline (30g), erythromycin (15 *μ*g), clindamycin (2 *μ*g), chloramphenicol (30 *μ*g), and gentamicin (10 *μ*g). Susceptibility pattern was interpreted by comparison of the zone of inhibition according to the Clinical and Laboratory Standards Institute (CLSI, 2014) guideline and reported as sensitive, intermediate, and resistant [24]. Standard strains of *S*. *aureus* (ATCC25923) were used as controls on the biochemical tests and agar plates including MHA with antimicrobial discs to assure the testing performance of antimicrobial discs.

Data were entered and analyzed using SPSS version 20 for Windows. Stepwise logistic regression model was considered to determine factors associated with wound infection. Adjusted odds ratio and 95% CI were calculated to measure the strength of the association. *p* values <0.05 were considered as statistically significant.

## 3. Results

### 3.1. Sociodemographic Characteristics of Study Participants

In this study, a total of 266 study participants were included. Of these, 180 (67.7%) were male and 86 (32.3%) were female. The mean ages of the study participants were 33.2 ± 17.8 years (range from 5 to 81 years). One-fourth of the study participants had no formal education, while the majorities were lived in urban (205, 77.1%) (Table 1).

### 3.2. Prevalence of MRSA

**Figure 1:**
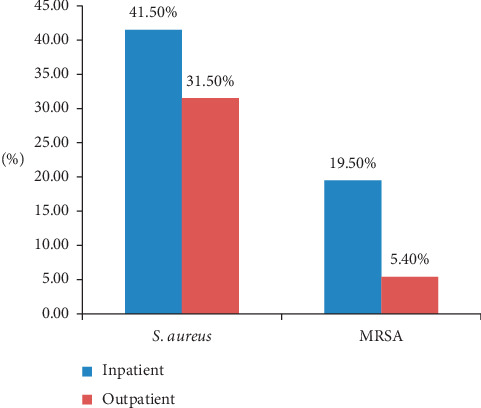
Distribution of *S. aureus* and MRSA among patients with wound infection in relationship with outpatients and inpatients in Dessie Referral Hospital.

Out of 266 patients suspected of developing wound infection, 92 (34.58%) have culture-confirmed *S. aureus* wound infections. Of these, 26 (28.3%) were MRSA. The overall prevalence of MRSA among the study population was 9.8% (26/266). Among 82 inpatients and 184 outpatients suspected of wound infection, 41.5% (34/82) and 31.5% (58/184) were culture-positive for *S. aureus*, respectively. The overall prevalence of MRSA in inpatients and outpatients was 19.5% (16/82) and 5.4% (10/184), respectively (Figure 1).

### 3.3. Antibiotic Resistance Pattern

Out of the 92 *S. aureus* isolated from wound swab including MRSA, 78 (84.8%) showed a high level of resistance to penicillin and 4 (4.3%) showed a low level of resistance to clindamycin while MRSA showed full (100%) resistance rate to penicillin followed by erythromycin and ciprofloxacin (16, 61.5%) and cotrimoxazole and gentamicin (14, 53.8%) (Table 2).

### 3.4. Multidrug Resistance Pattern of MRSA

In this study, a high prevalence of multidrug resistance (MDR) to MRSA was observed as compared to methicillin-sensitive *Staphylococcus aureus* (MSSA) which accounted for 18 (69.2%) and 2 (3%), respectively; none of the strains were resistant to all antibiotics tested. However, 10 (15.2%) MSSA were sensitive to all antibiotics tested (Table 3).

### 3.5. Bivariate and Multivariate Analysis of Factors Associated with MRSA among Wound Infection Individuals

In a bivariate logistic regression analysis, wound infection due to MRSA showed significant association with occupation, history of recent admission, history of recent surgery, being diagnosed in the inpatient department, and low body mass index (BMI) (<18.5). However, other factors such as age, sex, education, residence, history of previous antibiotic use, and chronic illness did not show statistically significant association.

In a multivariate logistic regression analysis, the abovementioned associated factors remained associated with wound infection due to MRSA except recent history of admission. Farmers were 6 times more likely to develop MRSA wound infection (AOR = 6.1; 95% CI (1.086–33.724)) than housewives. Patients who have low BMI were 13.9 times more likely to develop MRSA wound infection (AOR = 13.89; 95% CI (4.919–39.192)) than their counterparts. In addition, those inpatients were 3.6 times more likely to be infected with MRSA (AOR = 3.56; 95% CI (1.429–8.857)) as compared to those diagnosed in OPD (Table 4).

## 4. Discussion

Wound infection due to MRSA was a major concern in resource-limited countries, in particular, Ethiopia, where there are poor infection prevention and control measures [25]. In this study, the prevalence of *S. aureus* wound infection was 34.5%. This finding is in line with the study conducted in Debre Markos (39.7%) [18] and Cameroon (28.9%) [26]. On the other hand, this finding is higher than studies conducted in Jimma (23.6%) [19], Nigeria (26.6%) [27], Tanzania (26.7%) [28], and Brazil (20%) [29]. However, the prevalence reported in the current study is lower than a study reported in Addis Ababa (57.8%) [30] and Uganda (41%) [31]. The variation in prevalence might be due to variation in the study subjects, study conducted time, and the method employed for the detection of *S. aureus.*

In our study, the overall prevalence of MRSA was 9.8%. This finding is similar to the results reported from studies in Addis Ababa (13.2%) [20], Eretria (9%) [32], and Cameroon (13.16%) [26] and lower than the results reported from previous studies in Ethiopia such as Debre Markos (19.6%) [18] and Jimma (17.4%) [19] and other African countries: Uganda (41%) [31] and Libya (31%) [33]. On the other hand, this study finding is higher than study reports from Nigeria (5.8%) [27], Brazil (5.6%) [29], and Tanzania (4.3%) [28]. The observed high prevalence of MRSA in our study may be due to the high rate of certain antibiotics use either due to availability or cost-effectiveness issues.

Regarding the possible associated risk factors, MRSA wound infections were significantly associated with occupation (farmers), patients with low BMI, and those patients who are currently admitted (inpatient) as compared to their counterparts. This might be because farmers may not have knowledge of utilizing healthcare services; in addition, their occupation may expose them to wound infection and make them use antibiotics without prescription. High prevalence of MRSA in admitted patients may be attributed by resistant strain bacterial cross-contamination in health institutions. Patients who have low BMI had higher odds of developing wound infection due to MRSA. Healthy people may carry MRSA asymptomatically for long periods of time, but patients with compromised immune system are at a significantly greater risk of symptomatic infections [13, 14].

Concerning the antimicrobial resistance profile of the isolates, in the present study, *S. aureus* isolates showed resistance to penicillin (84.8%), gentamicin (15.2%) ciprofloxacillin (18.4%), clindamycin (4.3%), erythromycin (26.1%), cotrimoxazole (16.3%), doxycycline (9.7%), and chloramphenicol (8.7%). The resistance profile of *S. aureus* to penicillin in our study is similar to the results obtained within DRH (82.2%) [18]. In other studies, resistance to penicillin done in Tanzania [28] and Jimma, Ethiopia [34], is slightly higher and reported as 97% and 100%, respectively. The resistance to clindamycin in the current study is similar to other studies in Ethiopia in which the resistance is less than 50% [20, 30]. We noticed that the resistance rate of other antibiotics listed above is varied in different studies conducted in Ethiopia [18, 19].

The current study showed MRSA isolates were resistant to penicillin (100%), gentamicin (53.8%), ciprofloxacillin (61.5%), clindamycin (7.7%), erythromycin (61.5%), cotrimoxazole (53.8%), doxycycline (30.8%), and chloramphenicol (26.9%). Similarly, studies conducted in different areas showed MRSA isolates were 100% resistant to penicillin [18, 35]. In this study, the resistance of MRSA isolates to gentamycin is higher compared to the study done in Yekatit 12 Hospital in Addis Ababa (38.2%) [20]. Similarly, the resistance to ciprofloxacin is slightly higher compared to the results reported in Tanzania (54%) [28]. In contrast, the resistance to clindamycin, erythromycin, cotrimoxazole, and chloramphenicol is lower compared to the studies done in Ethiopia [18, 20].

The main variation in drug resistance patterns among different studies might be due to the indiscriminate use and availability of these antibiotics in a certain area. The variation of resistance rate among different areas indicates the resistance pattern of antibiotics varies according to regional and geographical location and also changes through time.

Furthermore, high prevalence of multidrug-resistant MRSA (69.2%) was reported in the study area. This finding is concordant with the study conducted in northern India whereby 73% of MRSA strains were multidrug-resistant [36]. Likewise, a study conducted in Debre Markos showed all MRSA strains isolated were resistant to ≥3 antibiotics [18]. High prevalence of multidrug resistance may predispose patients to infection with intractable isolates, emphasizing the need for improved infection control practices and guidelines for the use of antibiotics in this setting.

The current study results could not show MRSA is community-associated or healthcare-associated.

## 5. Conclusion

Out of 266 patients suspected with wound infection, 92 (34.58%) have culture-confirmed *S. aureus*. Of these, 26 (28.3%) were MRSA. Wound infection due to MRSA showed significant association with occupation, being diagnosed in the inpatient department, and body mass index. Greater than 50% of MRSA isolates were resistant to gentamicin, ciprofloxacillin, cotrimoxazole, and erythromycin.

## Figures and Tables

**Table 1:** Sociodemographic characteristics of patients with wound infections at Dessie Referral Hospital, Northeast Ethiopia.

Characteristics		Frequency	Percent
Age (years)	5–14	25	9.4
	15–24	83	31.2
	25–34	58	21.8
	35–44	31	11.7
	45–54	22	8.3
	55–64	28	10.5
	>64	19	7.1

Sex	Male	180	67.7
	Female	86	32.3

Educational status	No formal	67	25.2
	Primary	93	35
	Secondary	75	28.2
	College/university	31	11.7

Residence	Rural	61	22.9
	Urban	205	77.1

Occupation	Civil servant	47	17.7
	Farmer	30	11.3
	Merchant	29	10.3
	Housewife	26	9.8
	Daily labor	42	15.8
	Others	92	34.6

Patient setting	Inpatient	82	30.8
	Outpatient	184	69.2

*Note*. Others: jobless, beggar, student, and NGO.

**Table 2:** Antibiotic resistance pattern of *S. aureus* and MRSA from wound infection in Dessie Referral Hospital.

Antibiotics	Resistance pattern (%)	
	*S. aureus* (*N* = 92)	MRSA (*N* = 26)
Penicillin	78 (84.8%)	26 (100%)
Gentamicin	14 (15.2%)	14 (53.8%)
Ciprofloxacillin	17 (18.4%)	16 (61.5%)
Clindamycin	4 (4.3%)	2 (7.7%)
Cefoxitin	26 (28.3%)	26 (100%)
Erythromycin	25 (26.1%)	16 (61.5%)
Cotrimoxazole	15 (16.3%)	14 (53.8%)
Doxycycline	9 (9.7%)	8 (30.8%)
Chloramphenicol	8 (8.7%)	7 (26.9%)

**Table 3:** MDR pattern of bacteria isolated from wound infection among patients attending Dessie Referral Hospital.

*S. aureus* (*n* = 92)	Resistance pattern, *n* (%)							
	*R*0	*R*1	*R*2	*R*3	*R*4	*R*5	*R* ≥ 6	MDR (≥3)
MSSA (*n* = 66)	10 (15.2)	50 (75.8)	4 (6.1)	1 (1.5)	0	1 (1.5)	0	2 (3%)
MRSA (*n* = 26)	0	0	8 (30.8)	2 (7.7)	1 (3.8)	2 (7.7)	13 (50)	18 (69.2)

*Note*. *R*0, *R*1, *R*2, *R*3, *R*4, *R*5, *R*6, sensitive to all, resistance to one, two, three, four, five, and greater than six antibiotics tested, respectively; MDR (≥3): multidrug resistance (for greater than or equal to three antibiotics). MSSA: methicillin-sensitive *Staphylococcus aureus*; MRSA: methicillin-resistant *Staphylococcus aureus*.

**Table 4:** Bivariate and multivariate analysis of factors associated with MRSA among patients with wound infection at Dessie Referral Hospital.

Variable		MRSA		COR (95% CI)	AOR (95% CI)	*p* value
		Yes	No			
Age (years)	5–14	1	24	1	1	
	15–24	7	76	2.21 (0.259–18.9)	0.74 (0.066–8.329)	0.808
	25–34	3	55	1.309 (0.130–13.233)	0.881 (0.056–13.759)	0.926
	35–44	1	30	0.800 (0.048–13.466)	0.26 (0.008–8.371)	0.448
	45–54	3	19	3.79 (0.363–39413)	2.4 (0.121–47.993)	0.564
	55–64	6	22	6.545 (0.729–58.756)	1.179 (0.58–23.863)	0.915
	>65	5	14	8.571 (0.907–80.993)	2.54 (0.158–40.847)	0.511

Occupation	Housewife	2	24	1	1	
	CS	3	44	1.63 (0.181–2.835)	0.69 (0.110–4.429)	0.702
	Farmer	3	27	4.7 (1.099–19.817)^*∗*^	6.1 (1.086–33.724)	0.040
	Merchant	7	22	3.341 (1.093–10.216)^*∗*^	1.47 (0.187–11.625)	0.713
	DL	3	39	0.875 (0.174–4.379)	1.11 (0.175–7.001)	0.913
	Others	8	84	0.808 (0.203–3.211)	2.1 (0.429–9.939)	0.366

Admission	Yes	18	85	4.103 (1.712–9.830)^*∗*^	1.21 (0.117–12.604)	0.872
	No	8	155	1	1	

Surgery	Yes	16	82	3.063 (1.330–7.054)^*∗*^	1.94 (0.540–6.950)	0.311
	No	10	158	1	1	

Patient setting	Inpatient	16	66	4.218 (1.833–9.765)^*∗*^	3.56 (1.429–8.857)	0.006
	Outpatient	10	174	1	1	

Chronic infection	Yes	6	29	2.2 (0.810–5.883)^*∗*^	2.1 (0.577–7.521)	0.263
	No	20	211	1		

BMI	<18.5	21	52	15.2 (5.462–42.216)^*∗∗*^	13.89 (4.919–39.192)	<0.001
	>18.5	5	188	1	1	

MRSA: methicillin-resistant *Staphylococcus aureus*; COR: crude odds ratio; AOR: adjusted odds ratio; DL: daily labour; CS: civil servant; BMI: body mass index. ^*∗*^*p* value < 0.05; ^*∗∗*^*p* value < 0.001.

## Data Availability

The data used to support the results of this research are available from the corresponding author upon request.

## Abbreviations

BMI: Body mass index
CA-MRSA: Community-associated methicillin-resistant *Staphylococcus aureus*
CoNS: Coagulase-negative *Staphylococcus aureus*
HA-MRSA: Healthcare-associated methicillin-resistant *Staphylococcus aureus*
MDR: Multidrug resistance
MRSA: Methicillin-resistant *Staphylococcus aureus*
MSSA: Methicillin-sensitive *Staphylococcus aureus*.
